# Combined Molecular Hydrogen and Vitamin C Treatment in Irradiated Normal and Cancer Cell Models: An Exploratory In Vitro Study and Case Reports

**DOI:** 10.3390/medsci14050580

**Published:** 2026-09-17

**Authors:** Michiko Miyakawa, Songbai Zhang, Jinan Behnan, Yu Ivy Zhang, Claudia Morganti, Keisuke Ito, Pauline Isakson, Per Uhlén, Ayako Miyakawa

**Affiliations:** 1 Department of Medical Biochemistry and Biophysics, Karolinska Institutet, Solnavägen 9, 171 77 Stockholm, Sweden; songbai.zhang@ki.se (S.Z.); jinan.behnan@einsteinmed.edu (J.B.); yu.zhang.2@ki.se (Y.I.Z.);; 2 Faculty of Sustainability Studies, Hosei University, 2-17-1 Fujimi Chiyoda, Tokyo 102-8160, Japan; 3 The Leo M. Davidoff Department of Neurological Surgery, Albert Einstein College of Medicine, Montefiore Medical Center, Bronx, NY 10467, USA; 4 Ruth L. and David S. Gottesman Institute for Stem Cell and Regenerative Medicine Research, Departments of Cell Biology and Medicine, Albert Einstein College of Medicine, Bronx, NY 10461, USA; claudia.morganti@einsteinmed.edu (C.M.); keisuke.ito@einsteinmed.edu (K.I.); 5 Clinical Immunology and Transfusion Medicine, Sahlgrenska University Hospital, 413 45 Gothenburg, Sweden; pauline.isakson@vgregion.se; 6 Division of Urology, Karolinska University Hospital, 171 64 Solna, Sweden

**Keywords:** ascorbate, molecular hydrogen, reactive oxygen species, radiation-induced toxicity, epithelial–mesenchymal transition, case report

## Abstract

**Background:** Radiation therapy is widely used to treat cancer but frequently causes normal tissue toxicities that compromise patients’ quality of life, highlighting the need for strategies that protect healthy tissue without reducing antitumor efficacy. Here, we investigated whether a redox-modulating intervention combining vitamin C and molecular hydrogen could mitigate radiation-induced injury while preserving or enhancing anticancer effects. **Methods:** Normal endothelial cells together with breast cancer and glioma cell lines were treated with vitamin C and hydrogen before and/or after irradiation, followed by assessments of cell viability, apoptosis, reactive oxygen species (ROS), and epithelial–mesenchymal transition (EMT)-associated gene expression. To explore the potential clinical relevance of these findings, two patients undergoing radiotherapy received the combination treatment as supportive care. **Results:** In normal cells, the combination treatment increased post-irradiation viability and reduced intracellular ROS levels, consistent with a cytoprotective antioxidant effect. In contrast, cancer cells exhibited reduced viability and increased apoptosis following the combined treatment, effects that were further enhanced by irradiation. In glioma cells, the combination treatment also suppressed the expression of EMT-associated genes. In the two clinical cases, improvements in radiation-associated toxicities were observed, including dermatitis, oral mucositis, gastrointestinal symptoms, and hair loss, along with improved treatment tolerability. **Conclusions:** Collectively, these exploratory findings suggest that the combination of vitamin C and hydrogen produces distinct responses among the cell models examined, with increased viability and reduced ROS in HUVECs and reduced viability, increased apoptosis, and altered EMT-associated gene expression in the cancer cell lines. These findings warrant further investigation of this combination as a potential adjunct to radiotherapy.

## 1. Introduction

Cancer is responsible for nearly 10 million deaths worldwide each year. Radiotherapy is a cornerstone of cancer treatment and is administered to millions of patients annually. However, despite its clinical benefits, radiotherapy is frequently associated with significant toxicities, including mucositis, dermatitis, xerostomia, gastrointestinal complications, bone marrow suppression, and radiation pneumonitis [1,2,3]. These adverse effects can substantially impair patients’ quality of life, limit treatment intensity, and increase healthcare costs. Although numerous radioprotective strategies have been investigated, few have been successfully translated into routine clinical practice, and the prevention and management of radiation-induced injury remain major clinical challenges [4]. Beyond cancer therapy, the widespread use of ionizing radiation in medical imaging, particularly computed tomography (CT), has further increased awareness of the potential risks associated with radiation exposure, emphasizing the need for safe and effective radioprotective interventions [5].

Ionizing radiation-induced tissue injury is largely mediated by reactive oxygen species (ROS), particularly hydroxyl radicals generated by the radiolysis of water [6]. Antioxidants can neutralize ROS [7,8], and their potential to reduce radiation-induced tissue damage has been extensively investigated [7,8,9,10]. However, this approach remains controversial because antioxidants may also protect tumor cells, potentially reducing the effectiveness of radiotherapy or promoting tumor progression [11,12]. Indeed, some randomized clinical trials have reported reduced radiotherapy efficacy and increased mortality associated with certain antioxidant supplements, including α-tocopherol and β-carotene [13,14,15]. Although amifostine is approved by the U.S. Food and Drug Administration (FDA) for radioprotection [16,17], its clinical use has been limited by adverse effects and variable clinical benefit [18]. Despite decades of research, few radioprotective interventions have been widely adopted in routine clinical practice. This gap underscores the need for safe, well-tolerated, and accessible interventions that protect normal tissues without compromising the therapeutic efficacy of radiotherapy.

Against this background, vitamin C and molecular hydrogen have emerged as promising, readily accessible radioprotective candidates with antioxidant and anti-inflammatory properties [19,20,21,22,23,24,25,26,27,28,29,30,31,32,33,34]. Compared with other treatment options, both are inexpensive, easy to administer, and generally well tolerated [35,36,37,38,39,40,41], making them attractive candidates for clinical investigation. Importantly, accumulating evidence suggests that they do not compromise, and may in some settings enhance, the anticancer effects of radiotherapy [42,43,44]. High-dose vitamin C has been shown to induce apoptosis and enhance radiosensitivity in several tumor types, and both agents have demonstrated anticancer activity in preclinical studies [42,43,44,45,46,47,48,49,50,51,52,53,54,55,56,57,58,59,60,61,62,63,64,65]. Nevertheless, whether combined administration of vitamin C and hydrogen can simultaneously protect normal tissues while preserving or enhancing antitumor efficacy remains unclear. In this study, we investigated whether a redox-modulating intervention combining vitamin C and hydrogen could produce distinct responses in irradiated endothelial and cancer cell models. To provide preliminary clinical context, we also report observations from two patients undergoing radiotherapy who received the combination treatment as supportive care. These uncontrolled observations suggest that adjunctive treatment with vitamin C and hydrogen may attenuate acute radiation-induced side effects.

## 2. Materials and Methods

### 2.1. Cell Lines

Human umbilical vein endothelial cells (HUVECs; ATCC, Manassas, VA, USA) were cultured in Endothelial Cell Growth Medium-2 (PromoCell, Heidelberg, Germany, Cat. C-22011) supplemented with Growth Medium-2 SupplementMix (PromoCell, Cat. C-39216) according to the manufacturer’s instructions. The human triple-negative breast cancer cell line MDA-MB-231 (ATCC) was maintained in Dulbecco’s Modified Eagle Medium (DMEM; Gibco, Grand Island, NY, USA, Cat. 41965) supplemented with 10% fetal bovine serum (FBS; Invitrogen, Carlsbad, CA, USA, Cat. 25149-079) and 1% Antibiotic-Antimycotic (Gibco, Cat. 15240-062), as previously described [66]. The murine glioma cell line GL261 was obtained from the National Cancer Institute at Frederick (Frederick, MD, USA) and expanded under serum-free sphere-forming conditions in DMEM/F12-GlutaMAX (MilliporeSigma, Burlington, MA, USA) supplemented with basic fibroblast growth factor (bFGF, 10 ng/mL), epidermal growth factor (EGF, 20 ng/mL; PeproTech, Rocky Hill, NJ, USA), and B27 supplement (1:50; STEMCELL Technologies, Vancouver, BC, Canada), as previously described [67]. All cell lines were maintained at 37 °C in a humidified incubator with 5% CO_2_.

### 2.2. Vitamin C and Molecular Hydrogen Treatment

The treatment protocols for vitamin C and molecular hydrogen were established based on previous in vitro studies [51,57] and optimized through preliminary experiments. MDA-MB-231 cells were treated with vitamin C at concentrations of 0.2 or 1 mM. For experiments assessing EMT-associated gene expression in GL261 glioma cells, higher vitamin C concentrations (5 and 10 mM) were used.

Molecular hydrogen (H_2_) was generated using a Pure Hydrogen Gas Generator (Model HB-H10). Culture medium was transferred to 30 mL tubes and bubbled with 99.99% H_2_ gas at a flow rate of 250 mL/min for at least 60 min, resulting in a dissolved hydrogen concentration exceeding 1000 ppb. The hydrogen-enriched medium was immediately transferred to cell culture dishes and used for subsequent cell treatment.

### 2.3. Radiation Treatment

Cells were irradiated with a single dose of 4 Gy using an Xstrahl irradiator (Xstrahl Life Sciences, Suwanee, GA, USA). The radiation dose was selected based on previous in vitro studies using the same cell lines [68]. HUVECs, MDA-MB-231, and GL261 cells were treated with vitamin C at the indicated concentrations, H_2_, or a combination of vitamin C and H_2_. Cells maintained in standard culture medium without vitamin C or H_2_ served as untreated controls. To evaluate the influence of treatment timing relative to irradiation, each treatment condition was applied according to one of four experimental schedules: (i) no irradiation, (ii) treatment before irradiation, (iii) treatment after irradiation, or (iv) treatment both before and after irradiation. All treatment conditions were evaluated under each experimental schedule in all three cell lines.

### 2.4. Viability Test

Cell viability was assessed 24 h after irradiation or, for non-irradiated controls, 24 h after treatment initiation. Viable and non-viable cells were quantified by Trypan Blue exclusion using a Countess Automated Cell Counter (Invitrogen, Life Technologies). Cell viability was calculated as the percentage of unstained (viable) cells relative to the total number of cells. Experiments were performed in triplicate for HUVECs and MDA-MB-231 cells and in quadruplicate for GL261 cells. Data are presented as mean values and were normalized to the untreated control, which was assigned a value of 1.

### 2.5. Detection of ROS

Intracellular reactive oxygen species (ROS) levels were assessed in HUVECs by flow cytometry using dihydroethidium (DHE; Invitrogen). Because DHE primarily detects superoxide-dependent oxidation, it was used as a surrogate marker of oxidative stress. Cells were analyzed under three experimental conditions: no irradiation, treatment initiated immediately after irradiation and maintained for 24 h, or treatment initiated 24 h before irradiation and continued for 24 h after irradiation. Single-cell suspensions were prepared from each experimental condition, and viable cells (1 × 10^6^ cells per sample) were incubated with DHE (1 µM) for 30 min at 37 °C. After washing with FACS buffer (PBS containing 4% FBS), samples were analyzed using an LSR II flow cytometer (BD Biosciences, San Jose, CA, USA), and data were processed with FlowJo v10 software (BD Biosciences).

### 2.6. Apoptosis Assay

Apoptosis was assessed in irradiated MDA-MB-231 breast cancer cells using a FAM-FLICA^®^ Caspase-3/7 Assay (Fluorescent-Labeled Inhibitor of Caspases; ImmunoChemistry Technologies, Davis, CA, USA) according to the manufacturer’s instructions. This assay detects activated caspase-3/7 in live cells using a cell-permeable fluorescent inhibitor that binds covalently to active caspases in apoptotic cells. Cells were assigned to one of four treatment conditions: irradiated control (culture medium only), irradiation plus vitamin C (1 mM), irradiation plus H_2_, or irradiation plus the combination of vitamin C (1 mM) and H_2_. Vitamin C and H_2_ treatments were initiated before irradiation and repeated after irradiation. Cells were then incubated with the FLICA reagent, washed to remove unbound probe, counterstained with Hoechst 33342, and imaged by confocal microscopy. For each treatment condition, fluorescence images were acquired from four predefined fields of view. Images were subsequently analyzed to quantify the percentage of caspase-3/7-positive nuclei, the normalized nuclear area positive for active caspase-3/7, and the normalized nuclear fluorescence intensity.

### 2.7. EMT Gene Expression Analysis

Total RNA was extracted from 1 × 10^6^ to 1.5 × 10^6^ GL261 cells using QIAzol reagent and the RNeasy Mini Kit (Qiagen, Germantown, MD, USA) according to the manufacturer’s instructions. RNA concentration and purity were determined using a NanoDrop spectrophotometer (Thermo Fisher Scientific, Waltham, MA, USA). First-strand cDNA was synthesized from 1 µg of total RNA using the iScript™ cDNA Synthesis Kit (Bio-Rad, Hercules, CA, USA).

Reverse transcription quantitative PCR (RT-qPCR) was performed using QuantiTect™ SYBR^®^ Green PCR Master Mix and pre-validated QuantiTect Primer Assays (Qiagen) on a QuantStudio™ 7 Flex Real-Time PCR System (Thermo Fisher Scientific). The PCR cycling conditions consisted of an initial activation step at 95 °C for 15 min, followed by 40 cycles of 94 °C for 15 s, 55 °C for 20 s, and 72 °C for 2 min. Each 20 µL reaction contained 10 µL of 2× QuantiTect SYBR Green Master Mix, 2 µL of 10× QuantiTect Primer Assay, and 20 ng of cDNA.

The following mouse primer assays were used: *Snai1* (Mm_Snai1_1_SG), *Snai2* (Mm_Snai2_1_SG), *Tgfβ1* (Mm_Tgfb1_1_SG), *Twist1* (Mm_Twist1_1_SG), *Stat3* (Mm_Stat3_1_SG), *Vim* (Mm_Vim_1_SG), *Cdh1* (Mm_Cdh1_1_SG), and *Ubc* (Mm_Ubc_1_SG).

All reactions were performed in technical triplicates. Samples with *Ct* values > 35 were excluded from further analysis. Relative gene expression was calculated using the 2^−Δ*Ct*^ method, where Δ*Ct* was calculated as the difference between the *Ct* value of the target gene and that of *Ubc*, which served as the reference gene.

### 2.8. Clinical Intervention (Hydrogen Inhalation and Vitamin C Administration)

Two patients undergoing radiotherapy who developed radiation-induced dermatitis provided written informed consent for publication of their clinical information and photographs. For the combined treatment, molecular hydrogen (H_2_) inhalation was initiated 10 min before intravenous vitamin C administration. High-dose vitamin C (25 g) was dissolved in 250 mL of infusion diluent and administered intravenously while patients simultaneously inhaled 99.99% H_2_ at a flow rate of 250 mL/min (H2JI1, Doctors Man Co., Ltd., Tokyo, Japan) through a nasal cannula.

During radiotherapy, the combined treatment was administered daily following each radiotherapy session. Patients were also encouraged to continue supportive treatment at home using liposomal vitamin C (Lypo-C), with the dose and frequency determined individually. Outside the radiotherapy period, the frequency of clinic-based hydrogen inhalation and intravenous vitamin C treatments varied according to clinical status, access to treatment facilities, and financial considerations, ranging from daily administration to once monthly.

### 2.9. Statistical Analysis

The Kruskal–Wallis test and Dunn’s multiple comparisons test were used to compare cell apoptosis and expression of EMT-related genes among different groups. Dunnett’s multiple comparisons test was used for comparing viability. Statistical analyses were performed using Microsoft Excel for Mac (v16.77), GraphPad Prism (v10.6.1; GraphPad Software, San Diego, CA, USA), and Jupyter Notebook (v6.5.4) with pandas library. Statistical significance was defined as *p* < 0.05.

## 3. Results

### 3.1. Experimental Assessment of the Effects of Vitamin C and Molecular Hydrogen on Cell Viability Following Irradiation

The effects of vitamin C and molecular hydrogen on cell viability following irradiation were assessed in normal endothelial cells (HUVECs) and two cancer cell lines (MDA-MB-231 and GL261). Five treatment conditions were examined: 0.2 mM vitamin C, 1 mM vitamin C, hydrogen treatment alone, hydrogen combined with 0.2 mM vitamin C, and hydrogen combined with 1 mM vitamin C. Treatments were administered either before irradiation, after irradiation, or both before and after irradiation. Cell viability was assessed 24 h after irradiation. As the present experiments assess short-term cell viability, they do not address the long-term proliferative capacity of surviving cells.

In normal HUVECs, all treatment conditions increased cell viability relative to untreated controls (Table 1). Although several treatments produced substantial increases in viability, statistical significance was reached only when cells received the combined treatment of 1 mM vitamin C and hydrogen both before and after irradiation (1.80-fold increase vs. control, *p* < 0.05). Notably, even in the absence of irradiation, vitamin C and hydrogen, administered either individually or in combination, consistently tended to increase cell viability, indicating that these treatments exert a beneficial effect on the metabolic activity and viability of normal endothelial cells independent of radiation exposure.

In MDA-MB-231 breast cancer cells, vitamin C and hydrogen, administered individually, generally reduced cell viability compared with untreated controls (Table 2). Following irradiation, cell viability was further decreased under all treatment conditions, with the exception of 0.2 mM vitamin C administered before irradiation, which produced a slight increase relative to the irradiated control. The combination of 1 mM vitamin C and hydrogen consistently produced the greatest reduction in cell viability. When administered before irradiation, the combination treatment reduced cell viability to 0.63-fold of the control (*p* < 0.05). Administration after irradiation or both before and after irradiation resulted in an even greater reduction, with cell viability decreasing to 0.44-fold of the control in both groups (*p* < 0.01 and *p* < 0.001, respectively).

In GL261 glioma cells, vitamin C and hydrogen also reduced cell viability at 24 h (Table 3). The greatest reduction was again observed with the combination of 1 mM vitamin C and hydrogen. Relative to untreated controls, this treatment reduced cell viability to 0.54-fold in non-irradiated cells (*p* < 0.001), to 0.73-fold when administered before irradiation (*p* < 0.05), and to 0.71-fold when administered after irradiation (*p* < 0.05). Unlike in MDA-MB-231 cells, administering the combination treatment both before and after irradiation did not further reduce cell viability and did not reach statistical significance.

Collectively, these findings show distinct treatment responses among the cell models examined. In HUVECs, the combination of 1 mM vitamin C and hydrogen produced the greatest increase in cell viability, particularly when administered both before and after irradiation. In MDA-MB-231 and GL261 cells, the same combination treatment was associated with reduced cell viability under several corresponding treatment conditions.

### 3.2. Mechanistic Evaluation of the Effects of Vitamin C and Molecular Hydrogen in Irradiated Cells

**Figure 1:**
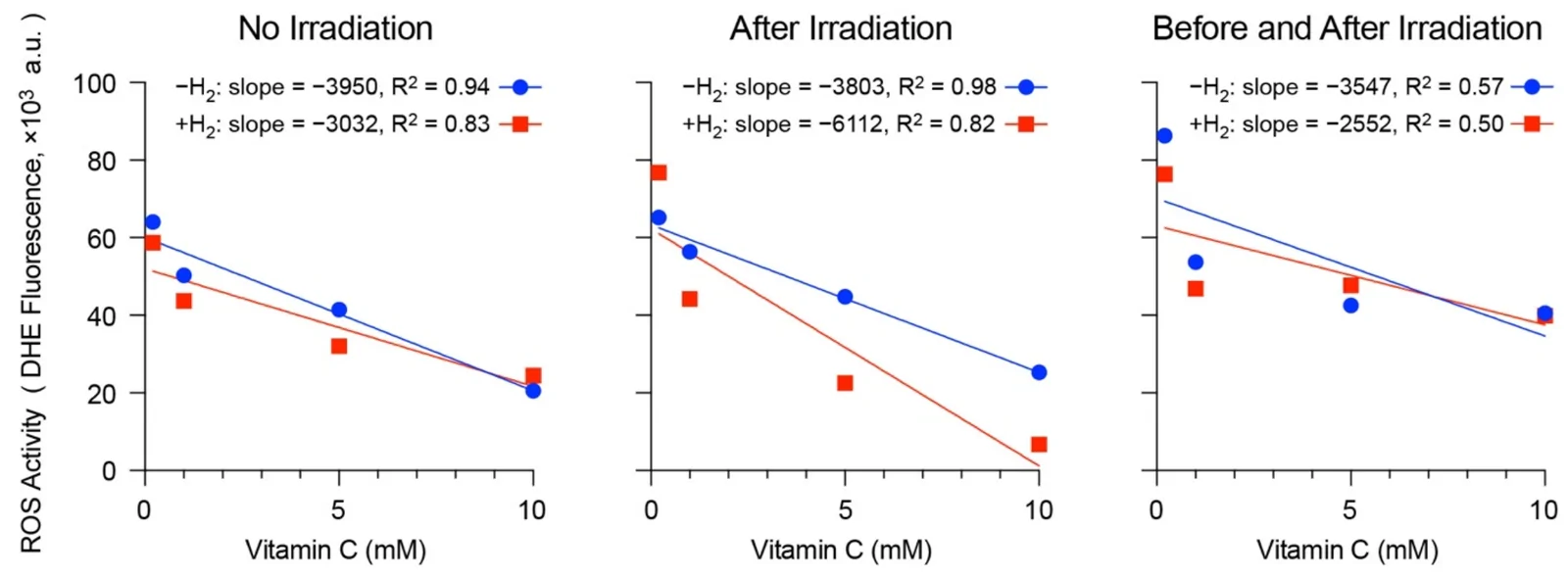
Concentration-dependent effects of vitamin C and molecular hydrogen on intracellular ROS levels in HUVECs. Intracellular reactive oxygen species (ROS) levels were assessed in HUVECs by dihydroethidium (DHE) fluorescence under three experimental conditions: no irradiation, treatment after irradiation, and treatment before and after irradiation. Cells were treated with vitamin C (0.2, 1, 5, or 10 mM) either alone (−H2) or in combination with molecular hydrogen (+H2). Linear regression was used to illustrate concentration-dependent trends, and the slopes and coefficients of determination (R2) are indicated. Each regression was based on four independent experiments corresponding to the four vitamin C concentrations (n = 4 data points per regression), with one measurement obtained at each concentration. The regression analyses are presented as descriptive trend analyses.

To gain mechanistic insight into the effects of vitamin C and molecular hydrogen, we examined intracellular reactive oxygen species (ROS) levels in HUVECs under three experimental conditions: non-irradiated cells, cells treated for 24 h immediately after irradiation, and cells treated for 24 h before and 24 h after irradiation (48 h total). Vitamin C was evaluated at concentrations of 0.2, 1, 5, and 10 mM, either alone or in combination with molecular hydrogen. Descriptive linear regression analysis revealed a concentration-dependent decrease in DHE fluorescence with increasing vitamin C concentrations under all three experimental conditions (Figure 1). Without irradiation, similar negative trends were observed with vitamin C alone (slope = −3950, R^2^ = 0.94) and in combination with molecular hydrogen (slope = −3032, R^2^ = 0.83). Following irradiation, negative concentration-dependent trends were observed for both vitamin C alone (slope = −3803, R^2^ = 0.98) and the combined treatment (slope = −6112, R^2^ = 0.82), with a steeper decline observed for the combination with molecular hydrogen. When treatment was administered both before and after irradiation, the concentration-dependent trends were less pronounced (vitamin C alone: slope = −3547, R^2^ = 0.57; combined treatment: slope = −2552, R^2^ = 0.50). These data indicate concentration-dependent trends toward reduced DHE fluorescence with increasing vitamin C concentrations, with the strongest negative trend for the combined vitamin C and molecular hydrogen treatment following irradiation.

To determine whether the reduction in cell viability following combined irradiation and treatment with vitamin C and/or hydrogen was associated with apoptosis, caspase-3/7 activation was assessed in MDA-MB-231 breast cancer cells. The assay was performed using the treatment regimen that produced the greatest reduction in cell viability, namely administration of vitamin C and/or hydrogen both before and after irradiation. A concentration of 1 mM vitamin C was selected because this concentration was already associated with reduced intracellular ROS levels following irradiation (as observed in Figure 1). Four experimental conditions were examined: untreated control (culture medium only), 1 mM vitamin C, hydrogen treatment alone, and the combination of 1 mM vitamin C and hydrogen.

**Figure 2:**
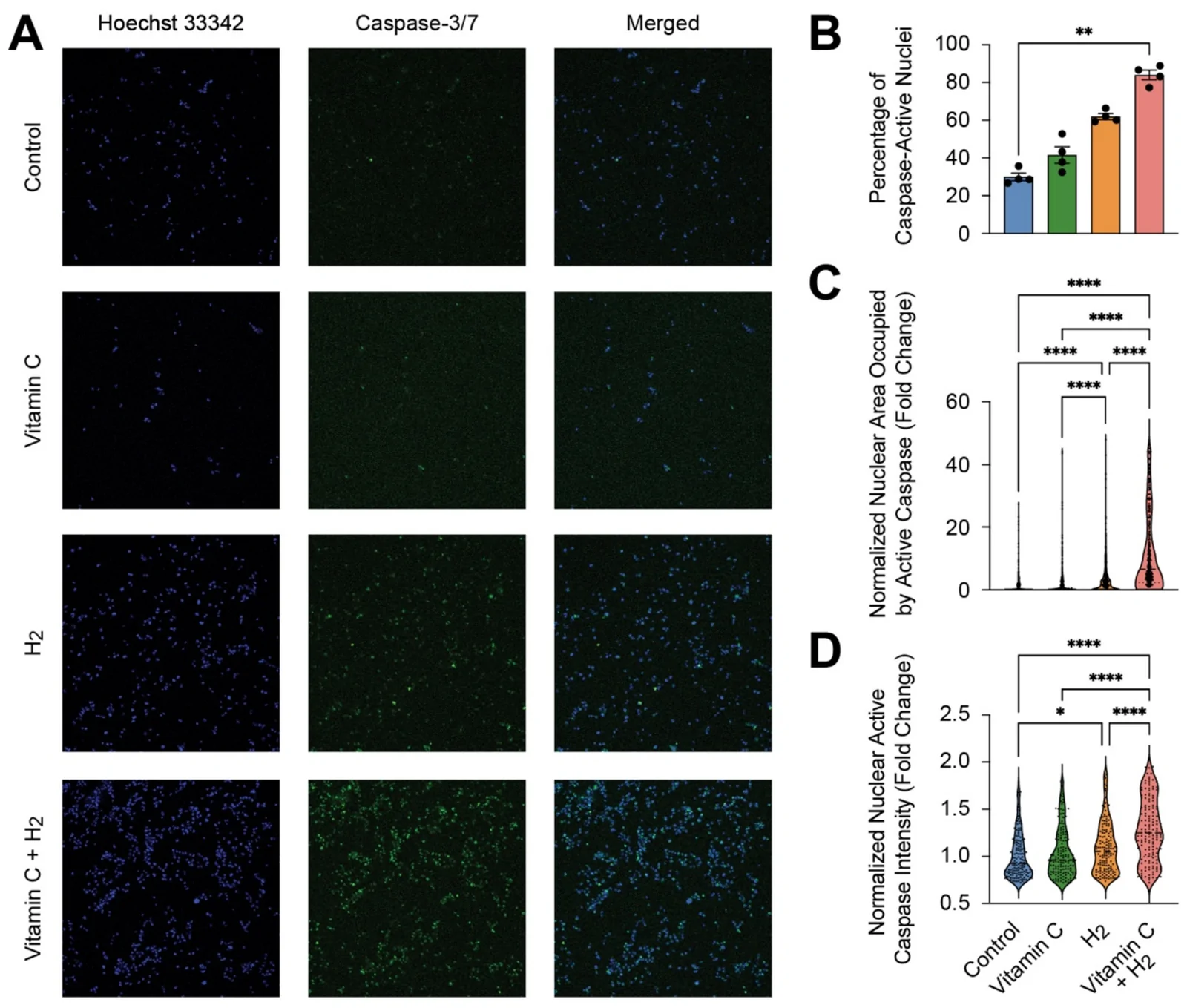
Assessment of apoptosis in irradiated MDA-MB-231 breast cancer cells following treatment with vitamin C and molecular hydrogen. (**A**) Representative confocal images of the FLICA apoptosis assay in irradiated MDA-MB-231 cells treated with irradiation alone (control), irradiation plus 1 mM vitamin C, irradiation plus molecular hydrogen (H_2_), or irradiation plus H_2_ combined with 1 mM vitamin C. Nuclei were counterstained with Hoechst 33342 (blue), and active caspase-3/7 was detected using the FLICA reagent (green). (**B**–**D**) Quantitative analysis of the percentage of caspase-3/7-positive nuclei ((**B**) *N* = 4), normalized nuclear area positive for active caspase-3/7 ((**C**) *N* = 4, *n* = 378), and normalized nuclear active caspase-3/7 fluorescence intensity ((**D**) *N* = 4, *n* = 150). The nuclear area positive for active caspase-3/7 quantifies the extent of the nuclear region exhibiting detectable caspase activity and provides a complementary measure of apoptosis alongside the proportion of caspase-positive nuclei and caspase fluorescence intensity. Data are presented as mean ± SEM. Violin plots show the distribution of individual measurements. Statistical significance was determined using the Kruskal–Wallis test followed by Dunn’s multiple comparisons test. * *p* < 0.05, ** *p* < 0.01, and **** *p* < 0.0001.

Minimal caspase-3/7 activation was detected in control cells (Figure 2A). Vitamin C alone produced a modest increase in caspase activation, whereas hydrogen treatment alone produced a more pronounced response. The combination of vitamin C and hydrogen resulted in the highest proportion of caspase-3/7-positive cells. Quantitative analysis showed that the combination treatment yielded the highest percentage of caspase-positive nuclei (Figure 2B), the largest nuclear area positive for active caspase-3/7 (Figure 2C), and the greatest nuclear caspase-3/7 fluorescence intensity (Figure 2D). The nuclear area positive for active caspase-3/7 provides a complementary measure of apoptosis by quantifying the extent of the nuclear region exhibiting detectable caspase-3/7 activity, alongside the proportion of caspase-positive cells and the intensity of the caspase signal. Together, these complementary measures indicate increased caspase-3/7 activation following the combination treatment and are consistent with the reduced cell viability observed in Table 2. These findings suggest that the reduction in viability following combined vitamin C and hydrogen treatment is associated with increased apoptotic cell death in irradiated MDA-MB-231 cells.

**Figure 3:**
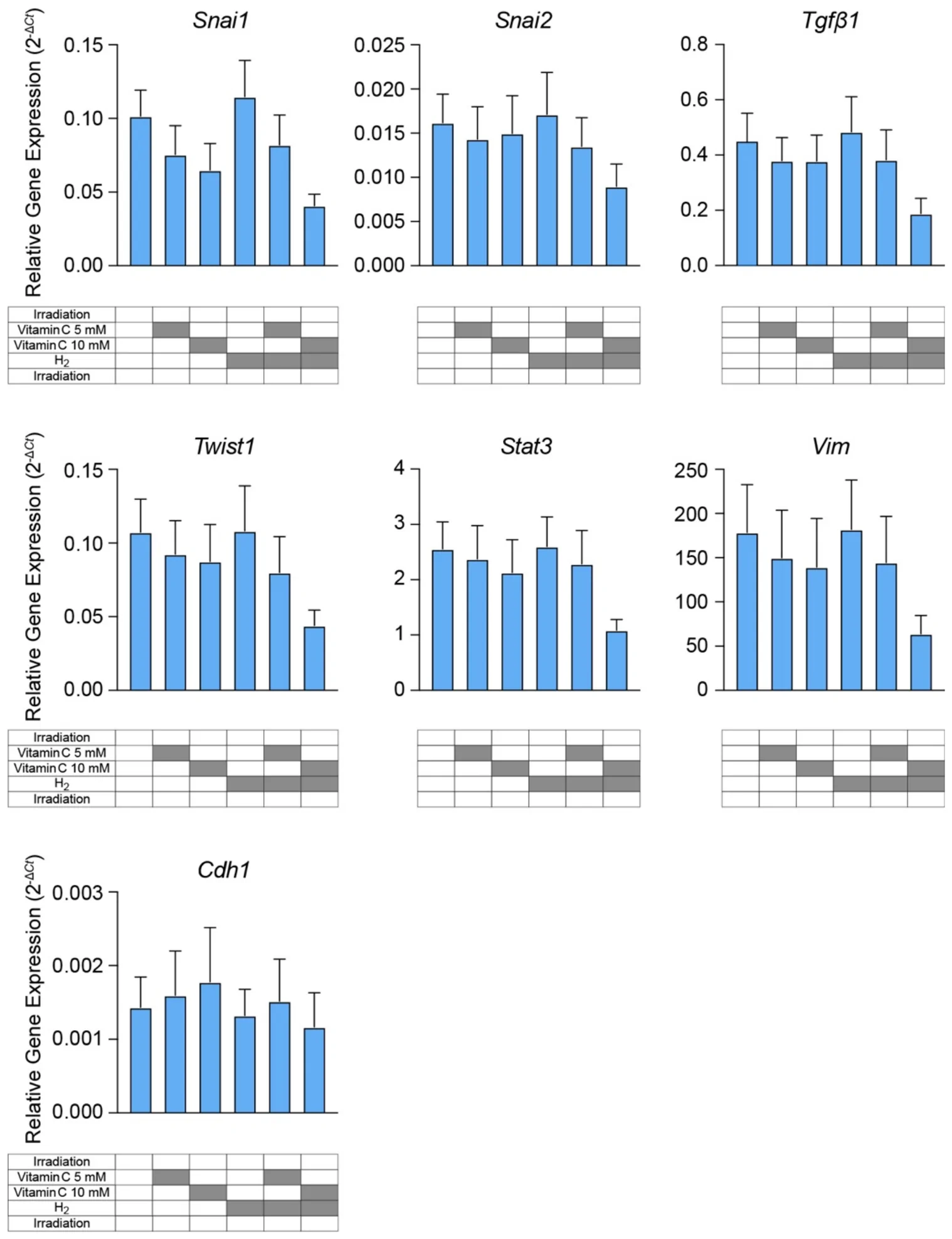
EMT-related gene expression in non-irradiated GL261 glioma cells following treatment with vitamin C and molecular hydrogen. Relative mRNA expression of the EMT-associated genes *Snai1*, *Snai2*, *Tgfβ1*, *Twist1*, *Stat3*, *Vim*, and *Cdh1* was quantified by RT-qPCR in non-irradiated murine GL261 glioma cells following the indicated treatments. Cells were treated with vitamin C (5 or 10 mM), molecular hydrogen (H_2_), or H_2_ combined with vitamin C. The treatment matrix beneath the graphs indicates the experimental condition corresponding to each bar. Each column in the matrix corresponds to the bars directly above it. Filled gray boxes indicate that the specified treatment was applied, whereas white boxes indicate that it was not applied. Combinations of filled boxes indicate combined treatments. Data are presented as mean ± SEM (*N* = 3). Statistical significance was assessed using the Kruskal–Wallis test followed by Dunn’s multiple comparisons test. *p* < 0.05.

In high-grade glioma, epithelial to mesenchymal transition (EMT) has been associated with therapy resistance and tumor progression [69]. To investigate whether vitamin C and hydrogen influence EMT-related transcriptional programs, the expression of seven EMT-associated genes (*Snai1*, *Snai2*, *Tgfβ1*, *Twist1*, *Stat3*, *Vim*, and *Cdh1*) was quantified in GL261 cells. In non-irradiated cells, treatment with vitamin C and hydrogen generally reduced the expression of the mesenchymal markers *Snai1*, *Snai2*, *Tgfβ1*, *Twist1*, *Stat3*, and *Vim*, with the most pronounced effects observed following treatment with 10 mM vitamin C combined with hydrogen (Figure 3). In contrast, expression of the epithelial marker *Cdh1* was only modestly affected by treatment.

**Figure 4:**
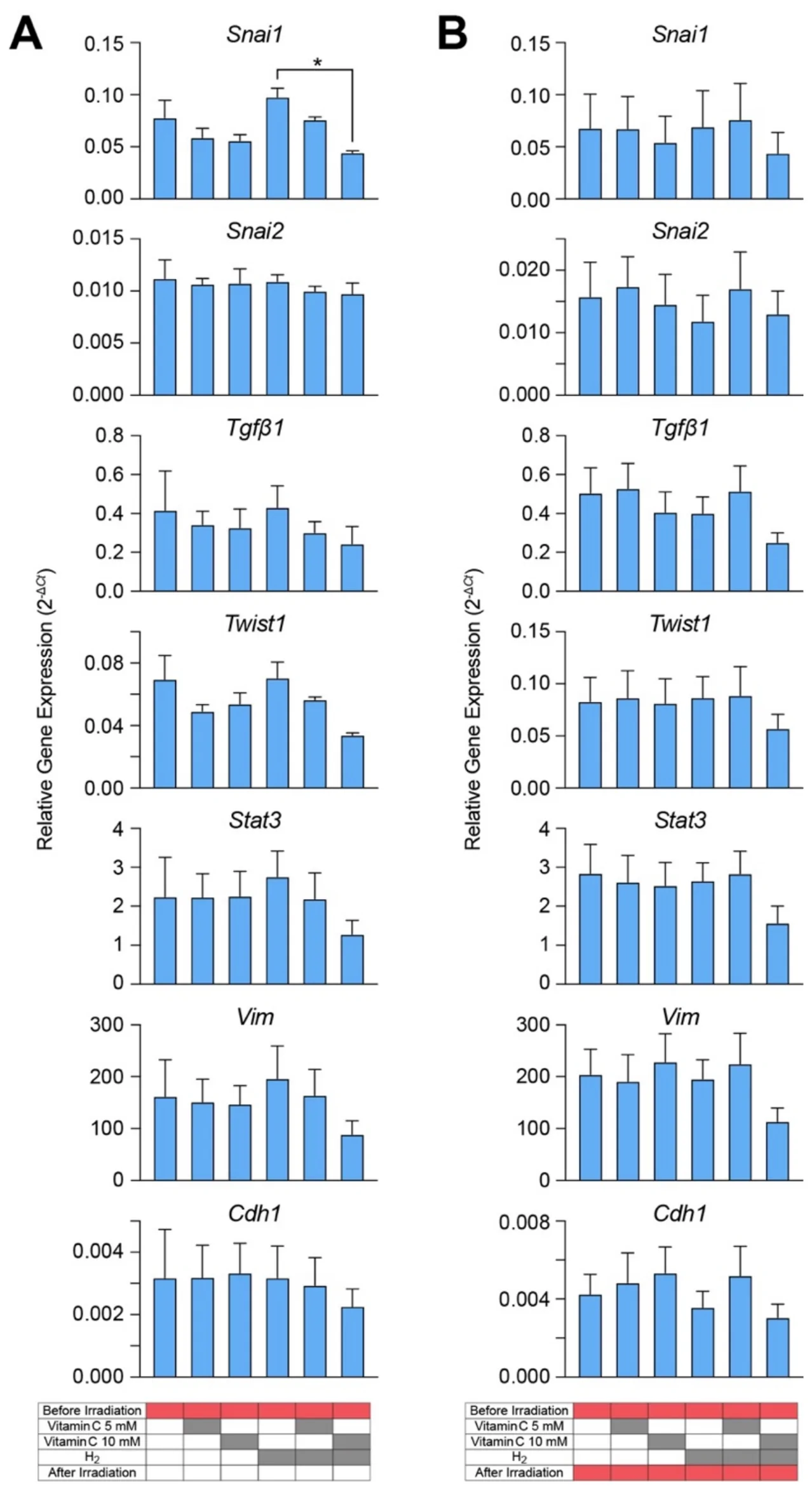
EMT-related gene expression in irradiated GL261 glioma cells following treatment with vitamin C and molecular hydrogen. Relative mRNA expression of the EMT-associated genes *Snai1*, *Snai2*, *Tgfβ1*, *Twist1*, *Stat3*, *Vim*, and *Cdh1* was quantified by RT-qPCR in irradiated murine GL261 glioma cells following the indicated treatments. (**A**) Treatments were administered after irradiation. (**B**) Treatments were administered both before and after irradiation. Cells were treated with vitamin C (5 or 10 mM), molecular hydrogen (H_2_), or H_2_ combined with vitamin C. The treatment matrix beneath the graphs indicates the experimental condition corresponding to each bar. Each column in the matrix corresponds to the bars directly above it. Filled gray and red boxes indicate that the specified treatment was applied, whereas white boxes indicate that it was not applied. Combinations of filled boxes indicate combined treatments. Data are presented as mean ± SEM (*N* = 3). Statistical significance was determined using the Kruskal–Wallis test followed by Dunn’s multiple comparisons test. * *p* < 0.05.

Following irradiation, combination treatment with vitamin C and hydrogen also reduced the expression of EMT-associated genes, with the strongest and most consistent inhibition again observed at the highest vitamin C concentration (10 mM) (Figure 4). Overall, 10 mM vitamin C combined with hydrogen suppressed the expression of multiple EMT-associated genes both in the absence and presence of irradiation.

### 3.3. Exploratory Clinical Application of Combination Therapy with Vitamin C and Molecular Hydrogen

Given our in vitro findings demonstrating differential effects of combined vitamin C and molecular hydrogen treatment in irradiated normal and cancer cells, we next explored the potential translational relevance of this approach in a clinical setting. High-dose intravenous vitamin C is used in many clinics worldwide as complementary supportive care for patients with cancer [70], although clinical responses vary considerably. Based on our experimental findings, we investigated whether the addition of hydrogen could serve as an adjuvant to vitamin C during radiotherapy, with the aim of supporting standard oncologic treatment and reducing radiation-associated side effects.

In this exploratory clinical application, two patients received high-dose intravenous vitamin C together with hydrogen inhalation following radiotherapy sessions as complementary supportive care. Treatment frequency was individualized according to clinical status, treatment schedule, and practical considerations, ranging from daily administration during radiotherapy to less frequent maintenance treatments. Both patients continued to receive standard-of-care oncologic therapy throughout the observation period.

In addition to clinic-based treatment, patients were encouraged, when feasible, to continue supportive therapy at home. Home hydrogen administration was not standardized and included hydrogen inhalation, hydrogen baths, hydrogen-enriched water, or hydrogen-generating supplements, depending on patient preference and equipment availability. Likewise, home vitamin C supplementation consisted of a liposomal formulation, with dose and frequency determined individually. Because these supportive interventions were self-funded, adherence and treatment intensity varied between the two patients.

#### 3.3.1. Case 1: 85-Year-Old Man

An 85-year-old man was diagnosed with stage IV malignant parotid carcinoma (salivary duct carcinoma). Following surgical resection, he received adjuvant chemotherapy and radiotherapy, with a total radiation dose of 60 Gy delivered in 40 fractions over 2 months. After each radiotherapy session, the patient underwent hydrogen inhalation combined with high-dose intravenous vitamin C (25 g diluted in 250 mL infusion solution). In addition, he used liposomal vitamin C and hydrogen inhalation at home as supportive therapy.

**Figure 5:**
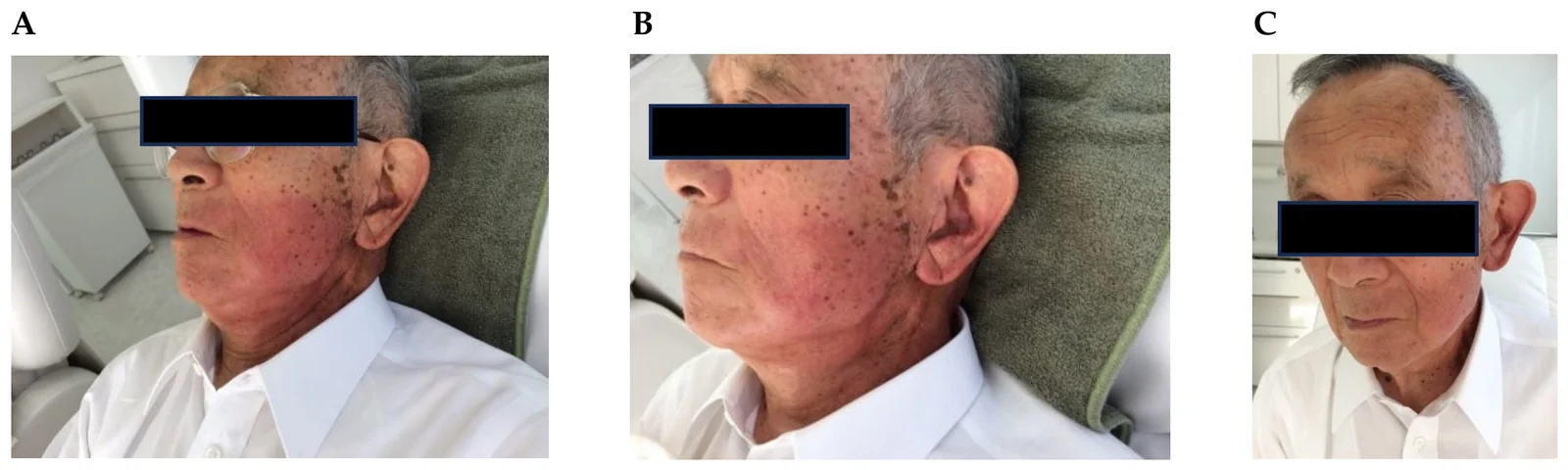
Clinical appearance of an 85-year-old patient with malignant parotid carcinoma during supportive treatment with vitamin C and molecular hydrogen following radiotherapy. (**A**) Appearance immediately before treatment, showing pronounced erythema and swelling following completion of radiotherapy. (**B**) Twenty minutes after treatment initiation. (**C**) At the end of the treatment session, showing marked improvement in the appearance of the irradiated skin.

Before initiation of the combined vitamin C and hydrogen treatment, the patient exhibited pronounced erythema, swelling, and inflammation within the irradiated field (Figure 5A). A visible reduction in erythema was observed within approximately 20 min after treatment initiation (Figure 5B). After 30 to 40 min, consisting of 10 min of hydrogen inhalation followed by approximately 20 to 30 min of concurrent intravenous vitamin C infusion, both erythema and edema appeared markedly reduced. By the end of the treatment session, the inflammatory reaction had largely resolved (Figure 5C). The patient also reported a noticeable reduction in pain during treatment.

#### 3.3.2. Case 2: 47-Year-Old Woman

A 47-year-old woman was diagnosed with nasopharyngeal carcinoma. She underwent definitive radiotherapy, receiving a total dose of 70 Gy in 35 fractions over 2 months in combination with systemic anticancer therapy. Radiotherapy was administered on an outpatient basis.

Throughout the treatment period, the patient performed daily supportive therapy at home, consisting of hydrogen gas inhalation, topical application of hydrogen mist to the irradiated skin, and daily supplementation with liposomal vitamin C.

**Figure 6:**
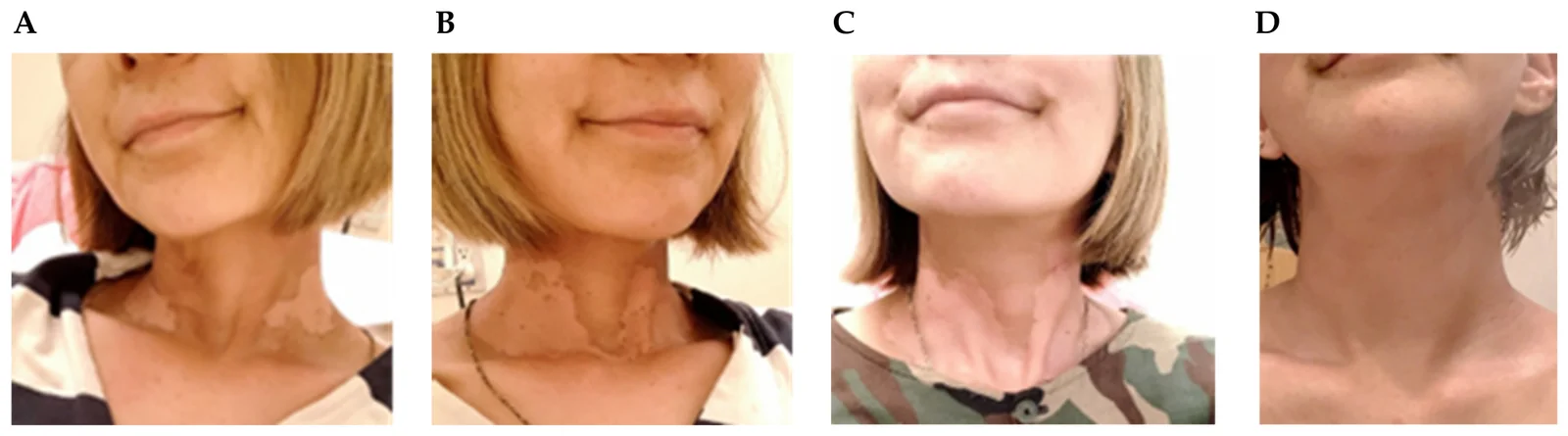
Clinical course of the irradiated neck in a 47-year-old patient with nasopharyngeal carcinoma during supportive treatment with vitamin C and molecular hydrogen throughout radiotherapy. (**A**) Seven days before completion of radiotherapy. (**B**) Five days before completion of radiotherapy. (**C**) On the final day of radiotherapy, showing only mild hyperpigmentation without severe radiation dermatitis. (**D**) One month after completion of radiotherapy, demonstrating resolution of the skin changes.

During radiotherapy, the patient developed only mild skin toxicity, characterized by limited hyperpigmentation and minimal desquamation. Seven days before completion of the 2-month course of radiotherapy, mild hyperpigmentation was evident within the irradiated field (Figure 6A). Similar skin changes were observed five days before the end of treatment (Figure 6B) and at completion of radiotherapy (Figure 6C), with no marked erythema or severe radiation dermatitis. According to the treating physician, the severity of the skin reaction appeared lower than typically expected for this treatment regimen. One month after completion of radiotherapy, the hyperpigmentation had largely resolved, and the skin appearance had returned close to baseline (Figure 6D).

Notably, both patients completed the two-month course of radiotherapy without developing severe oral mucositis, radiation dermatitis, gastrointestinal toxicity, or alopecia, all of which are common adverse effects associated with radiotherapy.

Although based on only two observational cases, these clinical findings suggest that combined vitamin C and hydrogen therapy may be a feasible supportive intervention during radiotherapy. The apparent reduction in acute radiation-induced toxicity, together with the favorable tolerability of the treatment, warrants further investigation in prospective controlled clinical studies to evaluate its safety, efficacy, and underlying mechanisms of action.

## 4. Discussion

### 4.1. Effects of Vitamin C

Vitamin C has long been investigated as a complementary agent in cancer therapy, beginning with the pioneering work of Cameron and Pauling in the 1970s [71]. As the principal water-soluble antioxidant in human plasma and mammalian cells, vitamin C is actively accumulated against a concentration gradient, highlighting its importance in maintaining intracellular redox homeostasis. In addition to scavenging reactive oxygen species (ROS), vitamin C has been reported to modulate gene expression, cellular differentiation, and oxidative DNA damage biomarkers [72]. Although randomized clinical evidence remains limited, animal studies and case reports suggest that high-dose vitamin C may attenuate radiation-induced normal tissue injury [19,20,22].

At pharmacological concentrations, vitamin C promotes extracellular hydrogen peroxide generation through metal-catalyzed oxidation [56]. Normal cells are generally protected from oxidative damage by catalase and peroxidase activity, whereas many cancer cells possess impaired antioxidant defenses, rendering them more susceptible to oxidative stress-induced apoptosis [73]. In conventional cell culture media, pharmacological concentrations of vitamin C (≥1 mM) readily generate extracellular H_2_O_2_ [51]. Exposure to 10 mM vitamin C is therefore likely to induce substantial oxidative stress under these experimental conditions, which may account for the marked suppression of EMT-associated gene expression observed in Figure 3 and Figure 4.

Our findings are consistent with the context-dependent antioxidant and pro-oxidant properties of vitamin C. In irradiated HUVECs, vitamin C, particularly at 1 mM and in combination with molecular hydrogen, was associated with reduced intracellular ROS levels and increased cell viability. In MDA-MB-231 breast cancer cells, vitamin C-containing treatments were instead associated with reduced viability and increased apoptosis. These differing responses may reflect cell-type-specific differences in redox regulation, although the present experiments were not designed to establish selective effects between tissue-matched normal and malignant cells. Furthermore, treatment with vitamin C, particularly when combined with hydrogen, suppressed the expression of multiple EMT-associated genes in GL261 glioma cells, suggesting that the treatment may also influence transcriptional programs associated with tumor progression and therapy resistance.

Interestingly, GL261 and MDA-MB-231 cells responded differently to the combination treatment. The greatest reduction in GL261 cell viability was observed in the absence of irradiation, whereas MDA-MB-231 cells exhibited the strongest response when vitamin C and hydrogen were administered together with irradiation. One possible explanation is that, in addition to inducing cell death, sublethal irradiation can activate pro-survival signaling pathways that promote cellular recovery and stress adaptation [74]. The balance between pro-apoptotic and pro-survival signaling is highly context dependent and varies among tumor types because of differences in their genetic, metabolic, and signaling characteristics. Such differences may underlie the distinct responses observed in GL261 and MDA-MB-231 cells.

Despite the ability of intravenous administration to transiently achieve pharmacological plasma vitamin C concentrations, clinical anticancer effects have remained inconsistent. This variability suggests that additional biological factors influence therapeutic efficacy and may explain why high-dose vitamin C has not been adopted as standard oncologic therapy. Our findings raise the possibility that modulation of cellular redox homeostasis with hydrogen may enhance the therapeutic profile of pharmacological vitamin C by simultaneously protecting normal cells while increasing the susceptibility of cancer cells to oxidative stress. This hypothesis warrants further investigation in preclinical models and prospective controlled clinical studies.

### 4.2. Effects of Molecular Hydrogen

Molecular hydrogen (H_2_), which is produced endogenously by intestinal bacteria, has attracted increasing interest as a therapeutic antioxidant because of its favorable safety profile and apparent selectivity for highly reactive oxygen species [75]. Hydrogen has been reported to preferentially neutralize hydroxyl radicals while largely sparing less reactive ROS involved in physiological redox signaling [76]. Owing to its small molecular size and high diffusibility, H_2_ readily crosses biological membranes and is thought to rapidly distribute throughout tissues, including mitochondria and the nucleus. This selective reactivity distinguishes hydrogen from many conventional antioxidants, which may indiscriminately suppress physiologically important redox signaling.

Experimental studies have demonstrated anti-inflammatory, anti-apoptotic, and cytoprotective effects of hydrogen in a wide range of disease models [77,78,79]. Early work showed that hydrogen reduced hydroxyl radical-mediated oxidative damage in cultured cells and in a rat model of ischemia-reperfusion injury without disrupting physiological ROS signaling [80]. More recent studies suggest that the biological effects of hydrogen extend beyond direct radical scavenging and involve modulation of redox-sensitive signaling pathways, gene expression, inflammation, and mitochondrial function [81]. Hydrogen has also emerged as a promising radioprotective agent. For example, Chuai et al. demonstrated radioprotective effects comparable to those of amifostine in experimental models, while avoiding the toxicity associated with amifostine [24]. Interestingly, hydrogen has also been reported to induce apoptosis or enhance radiation-induced cell death in several cancer models [55,62,63], suggesting that its biological effects are highly context dependent.

Our findings further support this context-dependent behavior. In irradiated HUVECs, hydrogen-containing treatments reduced intracellular ROS levels and improved cell viability, consistent with previously reported radioprotective effects in normal tissues. In contrast, the combination of hydrogen and vitamin C reduced viability and enhanced apoptosis in irradiated MDA-MB-231 breast cancer cells. In GL261 glioma cells, the same treatment also suppressed the expression of multiple EMT-associated genes, particularly when combined with high-dose vitamin C.

EMT is closely associated with tumor invasion, metastasis, and resistance to chemotherapy and radiotherapy [69]. The observed suppression of *Snai1*, *Snai2*, *Tgfβ1*, *Twist1*, *Stat3*, and *Vim* therefore suggests that combined hydrogen and vitamin C treatment modulates transcriptional programs associated with a mesenchymal phenotype. Whether these transcriptional changes translate into reduced invasion, metastasis, or treatment resistance remains to be determined. Nevertheless, the findings are consistent with an antitumor effect of the combined treatment and support further investigation of hydrogen as an adjunct to pharmacological vitamin C during radiotherapy.

### 4.3. Rationale for Combination Therapy

At pharmacological concentrations, vitamin C promotes extracellular hydrogen peroxide generation, which can subsequently give rise to highly reactive hydroxyl radicals through metal-catalyzed reactions [82,83].

Although this pro-oxidant activity is believed to contribute to the selective anticancer effects of pharmacological ascorbate, it may also increase oxidative stress in normal tissues. Molecular hydrogen has been proposed to selectively neutralize hydroxyl radicals while largely preserving reactive oxygen species involved in physiological signaling. Consequently, combining vitamin C with hydrogen may enhance the therapeutic window by protecting normal tissues from excessive oxidative damage while maintaining, or even enhancing, the anticancer effects of pharmacological vitamin C.

Our findings support this concept. In irradiated HUVECs, the combination of vitamin C and hydrogen reduced intracellular ROS levels and improved cell viability, consistent with a cytoprotective effect. In contrast, the same treatment reduced cell viability and enhanced apoptosis in irradiated MDA-MB-231 breast cancer cells, indicating increased radiosensitivity. In GL261 glioma cells, combination therapy suppressed the expression of multiple EMT-associated genes, suggesting modulation of transcriptional programs associated with tumor progression and therapy resistance. Together, these findings indicate that combined redox modulation can produce distinct biological responses among the cell models examined.

The mechanisms underlying these differential effects remain to be fully elucidated. The selective vulnerability of cancer cells to oxidative stress, together with differences in redox regulation, antioxidant capacity, and signaling pathways between normal and malignant cells, may contribute to the observed responses. Although these findings were obtained in a limited number of cell models and require validation in additional experimental systems, they provide a rationale for further investigation of combined vitamin C and hydrogen as an adjunct to radiotherapy.

### 4.4. Clinical Perspective

Although limited to two observational cases, our preliminary clinical findings suggest that combined hydrogen and vitamin C therapy is feasible and well tolerated as supportive care during radiotherapy. Both patients completed radiotherapy without severe acute toxicity, and the observed clinical responses were consistent with our in vitro findings demonstrating protection of normal cells while preserving, and potentially enhancing, cytotoxic effects in cancer cells. However, these observations should be interpreted with caution because they were uncontrolled, involved only two patients, and cannot establish causality or clinical efficacy.

Prospective controlled clinical studies are therefore required to determine whether combined hydrogen and vitamin C therapy can safely reduce radiation-induced toxicity without compromising antitumor efficacy. It will also be important to investigate whether this strategy may be applicable to other radiation-based treatments, including brachytherapy and radionuclide therapies such as radioiodine treatment.

## 5. Limitations

This study has several limitations. First, the experimental findings are based primarily on in vitro models and were complemented by only two uncontrolled observational clinical cases. Consequently, the clinical relevance of these findings should be interpreted with caution until confirmed in prospective controlled studies.

The in vitro analyses assessed acute cell viability rather than long-term clonogenic survival, which remains the gold standard for evaluating radiosensitivity. Furthermore, intracellular ROS levels were measured only in normal endothelial cells and not in the cancer cell lines. ROS measurements relied on dihydroethidium (DHE) fluorescence as a single indicator of oxidative stress and were not confirmed using complementary assays or direct measurements of specific reactive oxygen species. The stability of dissolved molecular hydrogen during the incubation period was not directly measured, and potential changes in H_2_O_2_ levels or pH resulting from vitamin C oxidation in the culture medium were not assessed. Therefore, we cannot exclude the possibility that changes in these parameters contributed to the observed cellular effects. Consequently, the relationship between ROS modulation and the observed biological effects remains associative rather than mechanistically established.

Additional limitations include the use of only one non-malignant cell type and two cancer cell lines, which may not fully capture the biological heterogeneity of normal tissues and human cancers. HUVECs are endothelial cells and are not tissue-matched normal counterparts of either MDA-MB-231 or GL261 cells. In addition, the different cell types required different culture media. Therefore, comparisons between HUVECs and the cancer cell lines should be interpreted as differences among the specific cell models examined rather than as definitive evidence of selective effects on normal versus malignant cells. Likewise, the observed changes in EMT-associated gene expression were not complemented by functional assays of cell migration or invasion, and therefore their biological significance requires further investigation.

Finally, the exploratory clinical observations were obtained under non-standardized treatment conditions. Access to clinic-based hydrogen inhalation and high-dose intravenous vitamin C varies considerably, as these interventions are often not reimbursed and may impose substantial out-of-pocket costs. Differences in treatment schedules, administration routes, and home-based supportive measures may therefore limit reproducibility and generalizability. Future prospective controlled clinical trials will be required to establish the safety, efficacy, optimal dosing, and timing of combined hydrogen and vitamin C therapy during radiotherapy.

## 6. Conclusions

In summary, combined treatment with vitamin C and hydrogen produced differential effects in the irradiated cell models examined. In normal endothelial cells, the combination reduced intracellular ROS levels and improved cell viability, whereas in MDA-MB-231 breast cancer and GL261 glioma cells it reduced cell viability, enhanced apoptosis, and suppressed EMT-associated gene expression. These findings suggest that combined redox modulation with vitamin C and hydrogen may produce cell-type-dependent responses. However, additional studies using tissue-matched normal and malignant cell models under comparable experimental conditions are required to determine whether the treatment selectively protects normal cells while preserving or enhancing anticancer effects.

Although these findings are encouraging, they should be interpreted with caution because the study was limited to one normal endothelial cell model, two cancer cell lines, acute viability assays, and two exploratory clinical observations. Consequently, the results should be regarded as preliminary and hypothesis generating. Further studies incorporating additional normal and cancer cell types, clonogenic survival assays, in vivo tumor models, and prospective controlled clinical trials are needed to determine whether this strategy can safely reduce radiation-induced normal tissue injury without compromising, or potentially improving, the therapeutic efficacy of radiotherapy.

## Figures and Tables

**Table 1:** Cell viability of HUVECs following irradiation and treatment with vitamin C and/or molecular hydrogen.

	Control	Vitamin C 0.2 mM	Vitamin C 1 mM	H_2_	H_2_ + Vitamin C 0.2 mM	H_2_ + Vitamin C 1 mM
No irradiation	1	1.31	1.35	1.21	1.04	1.14
Before irradiation	1	1.37	1.34	1.27	1.31	1.56
After irradiation	1	1.47	1.48	1.14	1.38	1.52
Before and after irradiation	1	1.52	1.58	1.09	1.61	1.80 *

Values are presented as mean (*N* = 3). H_2_, molecular hydrogen. The viability of the non-irradiated untreated control cells before normalization was 53%. Statistical significance was assessed using Dunnett’s multiple comparisons test versus the untreated control. * *p* < 0.05.

**Table 2:** Cell viability of MDA-MB-231s following irradiation and treatment with vitamin C and/or molecular hydrogen.

	Control	Vitamin C 0.2 mM	Vitamin C 1 mM	H_2_	H_2_ + Vitamin C 0.2 mM	H_2_ + Vitamin C 1 mM
No irradiation	1	0.94	0.81	0.84	0.88	0.85
Before irradiation	1	1.09	0.75	0.82	0.71	0.63 *
After irradiation	1	0.74	0.62 *	0.81	0.65 *	0.44 **
Before and after irradiation	1	0.72 *	0.62 **	0.76	0.76	0.44 ***

Values are presented as mean (*N* = 3). H_2_, molecular hydrogen. The viability of the non-irradiated untreated control cells before normalization was 62%. Statistical significance was assessed using Dunnett’s multiple comparisons test versus the untreated control. * *p* < 0.05, ** *p* < 0.01, and *** *p* < 0.001.

**Table 3:** Cell viability of GL261s following irradiation and treatment with vitamin C and/or molecular hydrogen.

	Control	Vitamin C 0.2 mM	Vitamin C 1 mM	H_2_	H_2_ + Vitamin C 0.2 mM	H_2_ + Vitamin C 1 mM
No irradiation	1	0.85	0.72 *	0.93	0.84	0.54 ***
Before irradiation	1	0.89	0.77 *	0.89	0.80	0.73 *
After irradiation	1	0.85	0.61 **	0.83	0.71 *	0.71 *
Before and after irradiation	1	0.90	0.77	0.92	0.88	0.76

Values are presented as mean (*N* = 4). H_2_, molecular hydrogen. The viability of the non-irradiated untreated control cells before normalization was 66%. Statistical significance was assessed using Dunnett’s multiple comparisons test versus the untreated control. * *p* < 0.05, ** *p* < 0.01, and *** *p* < 0.001.

## Data Availability

The original contributions presented in this study are included in the article. Further inquiries can be directed to the corresponding authors.
